# Two aspects of longevity are associated with rates of loss of telomeres in birds

**DOI:** 10.1002/ece3.9364

**Published:** 2022-10-10

**Authors:** F. Stephen Dobson, Quentin Schull, François Criscuolo

**Affiliations:** ^1^ University of Strasbourg, Institut Pluridisciplinaire Hubert Curien, UMR 7178 CNRS Strasbourg France; ^2^ Department of Biological Sciences Auburn University Auburn Alabama USA; ^3^ MARBEC, University of Montpellier, IFREMER IRD, CNRS Sète France

**Keywords:** body size, longevity, MCMCglmm, phylogenetic correlation, telomere dynamics

## Abstract

Telomeres, the terminal repetitive DNA sequences at the ends of linear chromosomes, have strong associations with longevity in some major taxa. Longevity has been linked to rate of decline in telomere length in birds and mammals, and absolute telomere length seems to be associated with body mass in mammals. Using a phylogenetic comparative method and 30 species of birds, we examined longevity (reflected by maximum lifespan), absolute telomere length, the rate of change in telomere length (TROC), and body mass (often strongly associated with longevity) to ascertain their degree of association. We divided lifespan into two life‐history components, one reflected by body size (measured as body mass) and a component that was statistically independent of body mass. While both lifespan and body mass were strongly associated with a family tree of the species (viz., the phylogeny of the species), telomere measures were not. Telomere length was not significantly associated with longevity or body mass or our measure of mass‐independent lifespan. TROC, however, was strongly associated with mass‐independent lifespan, but only to a much lesser degree at best with body mass‐predicted lifespan. Our results supported an association of TROC and longevity, in particular longevity that was independent of body size and part of the pace‐of‐life syndrome of life histories.

## Graphical abstract

We examined the association of telomere rate of change (TROC) with longevity among 30 bird species. Longevity has two components that were examined, one resulting from differences among species in body size and one representing the slow‐fast continuum in life histories. Slow‐fast pace of life was strongly associated with TROC, and body size had at best a poor association with TROC.

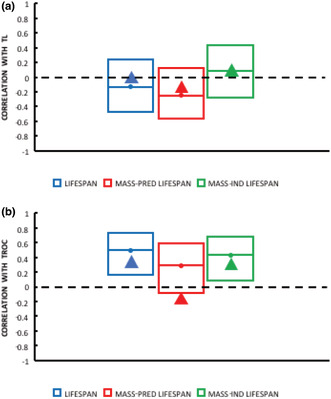

## 1 INTRODUCTION

Telomeres are repetitive nucleotide sequences at the terminal regions of eukaryote chromosomes. They serve to protect healthy chromosomes from DNA repair mechanisms that otherwise act on the terminal ends of chromosomes and organize the replication of DNA during cell division (de Lange, 2009). During replication, telomere sequences may be lost, thus shortening the telomere end (Olonikov, 1973). Telomerase may replace the lost sequences, thus lengthening it (Chan & Blackburn, 2004). Nonetheless, cells with over‐shortened telomeres become “senescent” or “self‐destruct” (termed apoptosis). When apoptosis occurs, the DNA‐encoded information of the cell is removed from the organism (Blackburn, 2000). Thus, telomeres may play a role in both cell senescence (or alternatively in cell immortalization due to the activity of telomerase; Tian et al., 2018), accumulation of senescent cells in organs (Campisi, 2005), and organismal senescence (Blasco, 2007; Young, 2018). Further, accelerated telomere loss at a given chronological age may indicate decreasing organismal condition, especially during early development, and may underlie both physiological stress and a shorter life (viz., advanced senescence; Bize et al., 2009; Boonekamp et al., 2014; Haussmann et al., 2003; Pepper et al., 2018; Sheldon et al., 2021; Sukyka et al., 2016; Whittemore et al., 2019).

Previous studies have suggested that due to the association of rate of telomere loss from chromosomes and organismal senescence, telomere dynamics during life are closely and functionally associated with lifespan among species of different body sizes and pace of life (e.g., Dantzer & Fletcher, 2015; Tricola et al., 2018). These studies primarily focused on birds and recognized the possible importance of body size and historical patterns (viz., the influence of phylogeny) in explaining a general pattern of relatively slowed loss of telomeres during life in larger species with syndromes of slower paces of life. These studies applied phylogenetic comparisons to 14 and 19 species of birds (TRF data), respectively, relatively small samples for robust phylogenetic analyses (though Dantzer & Fletcher, 2015 presented an analysis that assumed no significant phylogenetic associations). Tricola et al. (2018) suggested that the rate of telomere shortening exhibited a strong historical pattern that may have coevolved with lifespan. Both studies concluded that larger species with relatively slower life histories exhibited reduced rates of telomere shortening.

These key previous studies raised a series of questions that might be examined with a larger sample of species of birds. First, how flexible are telomere traits over phylogenetic history? Tricola et al. (2018) found that telomere length was not strongly influenced by the phylogenetic pattern, but telomere rate of change (TROC) was. Alternatively, Criscuolo et al. (2021) found that neither adult telomere length nor TROC showed a strong phylogenetic pattern in a corrected sample size of 52 bird species, a different result that needs to be explained.

Second, how do longevity, body size, and the pace of life interact with telomere dynamics? Dantzer and Fletcher (2015) found that all three variables covaried strongly with TROC, and TROC was lower for the longest‐lived bird species. The latter result was confirmed by Tricola et al. (2018) with or without body mass used as a covariate. In a phylogenetic comparative analysis of nine species of birds and mammals, Le Pepke and Eisenberg (2020) reported a low rate of telomere loss in long‐lived species but a trivial effect of body mass. These studies took a phylogenetic comparative approach where regressions were used to “control” for the phylogenetic patterns underlying variables. Still, none of them quantified how those phylogenetically controlled patterns of telomere dynamics change with lifespan at a given body size, that is, when influences of body size are statistically controlled (Udroiu, 2020).

Third, does telomere length per se change with species lifespan, with or without the influences of body size taken into account? Gomes et al. (2011) studied 61 species of mammals and found that adult telomeres were shorter in the longest‐lived species. Both body mass and longevity showed significant negative associations with telomerase activity and telomere length, respectively, independent of the phylogenetic pattern among the species (Gorbunova & Seluanov, 2009). The conclusion of Gomes et al. (2011) was that, for large species to evolve long lifespans, replicative aging occurred (i.e., short telomeres combined with repressed telomerase activity). A result confirmed by a re‐analysis of the same dataset recently (Le Pepke & Eisenberg, 2021), with emphasis on an inverse association of telomere length with body mass. These results suggest that short telomeres may have co‐evolved in long‐lived species as a consequence of body size, underlying the necessity for large species to control for higher risks of cell immortalization by a widespread cellular mechanism (Tian et al., 2018). Interestingly, the phylogeny‐adjusted analyses of Tricola et al. (2018) conducted in birds found no significant association of telomere length and longevity, whether or not influences of body mass were included. However, longevity and body mass are known to show strong covariance (Dantzer & Fletcher, 2015) and thus may be collinear. The analyses of more bird species by Criscuolo et al. (2021), however, found no significant association of adult telomere length and either body size or the pace of life, with or without inclusion of the phylogenetic pattern in the analyses, though they did not specifically examine longevity and its single‐trait association with telomere length.

The purpose of our present study was to examine associations of telomere length and TROC on the one hand, and body size and longevity on the other hand in birds. We did this using a restricted sample size of 30 of the bird species (see below for justification) reviewed in the comparative analysis of Criscuolo et al. (2021). We first asked whether any of the variables showed evidence of strong phylogenetic pattern, using the Bayesian meta‐analysis approach of Hadfield and Nakagawa (2010). Longevity and body size are closely associated in birds (e.g., Bennett & Owens, 2002; Criscuolo et al., 2021; Dantzer & Fletcher, 2015). Larger species reflect many aspects of life histories that covary over evolutionary time, in part because it takes longer to grow and survive to a large body size (Dobson, 2007). Further, bird species vary along a “slow‐fast continuum” that reflects alternative paces of life that are independent of body size (Gaillard et al., 1989). Thus, we examined variation in longevity that was strongly associated with body size (“mass‐predicted lifespan”), and longevity that was statistically independent of body size (“mass‐independent lifespan”). The latter reflects changes in lifespan that can be described as varying with the pace of life (Criscuolo et al., 2021; Dobson & Oli, 2007). Because differences between phylogenetically adjusted and unadusted associations of traits can be biologically informative (Price, 1997), we compared both of the aspects of lifespan to telomere length and TROC.

We also addressed a further issue with respect to how TROC is measured. First, our sample included estimates of TROC that used mean differences in telomere lengths between chicks and older birds (after Criscuolo et al., 2021). Such estimates have the advantage of including the chick period, when the greatest rates of telomere loss occur as birds age (Monaghan & Ozanne, 2018; Sidorov et al., 2009), but they have the disadvantage of including individuals that do not survive to adulthood (Dantzer & Fletcher, 2015). A bias may thus occur between the samples of younger and older birds. Thus, we estimated TROC only from samples of adult birds. A further problem is that some estimates of telomere length measure only DNA sequences of the terminal telomere repeats, whereas other methods include DNA sequences from the body of the chromosome (Remot et al., 2021). We restricted our analyses to those studies that used the former methods and thus produced the best estimates of telomere lengths.

## 2 MATERIAL AND METHODS

Our earlier study examined associations between telomere variables and aspects of life history for bird species (Criscuolo et al., 2021). Our comparative analysis followed the recommendations of the preferred reporting items for systematics reviews and meta‐analysis (PRISMA) statement (Liberati et al., 2009).

In the present study, we focused on 30 species for which adult telomere length, TROC, and life history variables were all recorded. For these species, telomere lengths were estimated in kilobases (kb) using electrophoretic separation of telomere restriction fragments or by quantitative fluorescent in situ hybridization (respectively, TRF and Q‐FISH; Remot et al., 2021). All studies used samples of erythrocytes. TROC was measured as the slope of telomere length regressed over age (kb/year) for each of the species (Haussmann et al., 2003; Tricola et al., 2018), but excluding the telomere lengths of hatchling chicks or yearlings (ages of 0–1 year). We followed Dantzer and Fletcher (2015) and Tricola et al. (2018) in using maximum lifespan in nature to typify longevity and mean adult female body mass to typify body size, and log‐transformed both variables. Among the 30 species of birds, body‐size‐independent aspects of longevity (longevity associated with the pace of life) were estimated as the residuals of maximum lifespan regressed on mean female body mass, and these residuals were checked for normality using a Q‐Q plot and Shapiro–Wilk test. When multiple sources were available, a mean value of adult telomere length or TROC was used.

We divided lifespan into two statistically independent parts, based on the regression of lifespan onto body size (as estimated by mean adult female body mass for each species). The first variable represented mass‐associated aspects of lifespan, as indicated by the predicted values of lifespan from the regression, and termed “mass‐predicted lifespan.” The residuals from the analysis were termed “mass‐independent lifespan,” and these two measures of lifespan were statistically uncorrelated. The latter variable estimated lifespan at a given body size and may be interpreted as a measure of lifespan on the slow‐fast continuum of the pace of life (Dobson & Oli, 2007; Gaillard et al., 1989). Associations of telomere and lifespan variables were examined by comparison of both correlation and by phylogeny‐adjusted correlation (after Price, 1997).

**FIGURE 1:**
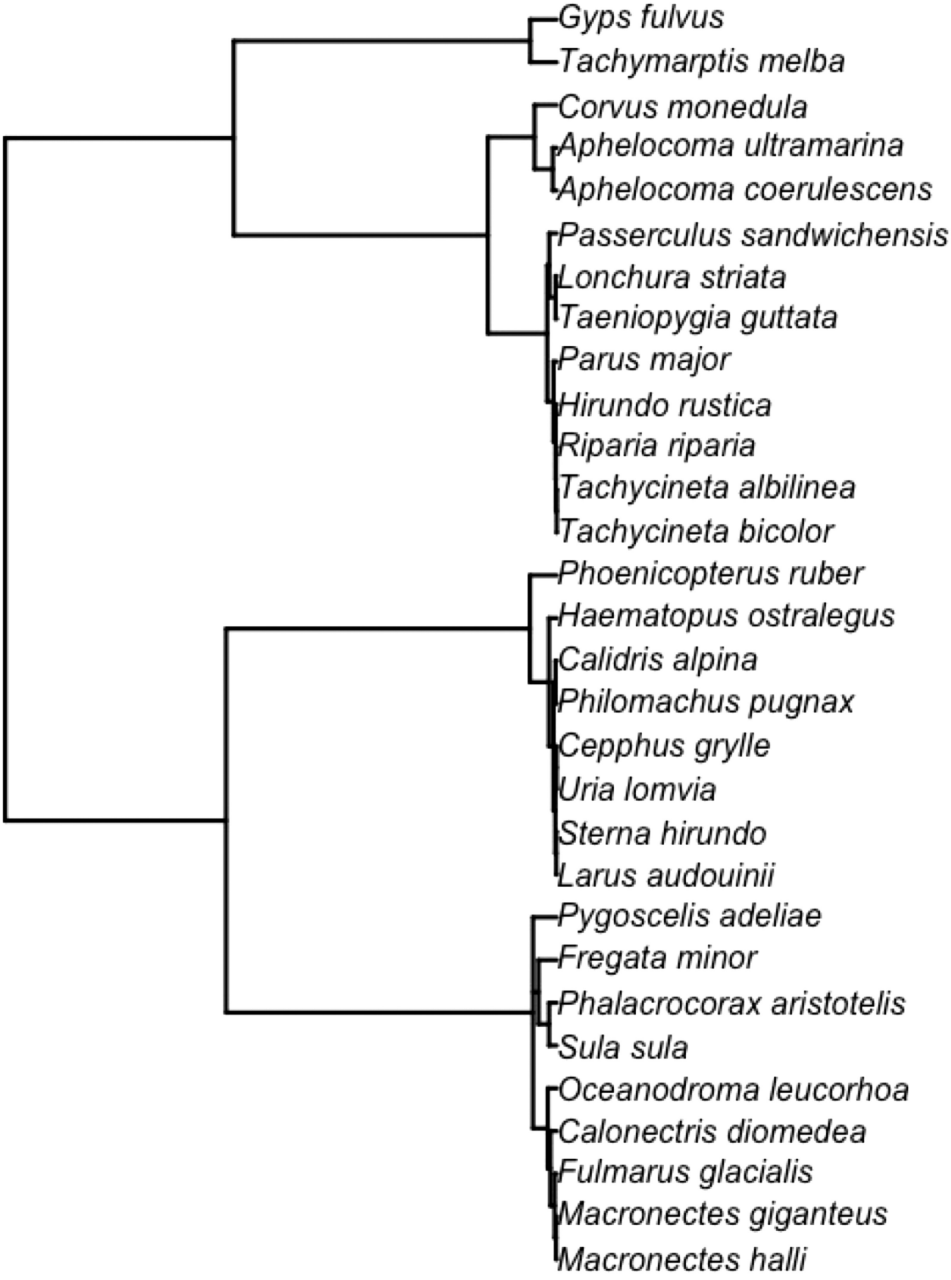
Phylogenetic tree of the 30 bird species for which adult telomere length and telomere length rate of change (TROC) were collected from published papers on avian telomeres using the TRF or Q‐FISH methodologies. The consensus phylogenetic tree was obtained from BirdTree.org (100 trees).

A phylogeny was obtained from BirdTree (Figure 1), with 100 phylogenetic trees downloaded from http://www.bird.tree.org (de Magalhaes & Costa, 2009; Jetz et al., 2012) using ape (Paradis & Schliep, 2019), apTreeshape (Orme et al., 2018), and caper R packages (Bortolussi et al., 2020). Branch lengths were estimated using the coalescent method, thus reflecting an estimate of relative divergence times for the phylogeny (Rannala & Yang, 2003). Associations of the bird phylogeny with adult telomere length, TROC, adult female body mass, maximum lifespan, and the residuals of lifespan on body mass were estimated using Hadfield and Nakagawa's (2010) Markov chain Monte Carlo generalized linear mixed model (MCMCglmm; Hadfield, 2010) package in R (R core team, 2020). The MCMCglmm package was also used to produce phylogeny‐adjusted estimate of associations of telomere, body mass, and longevity variables.

MCMCglmm was used to produce two types of results: (1) an estimate of the phylogenetic pattern in each of the study variables (viz., ρ = the proportion of variance that could be statistically accounted for by a matrix of the phylogenetic pattern); and (2) degree of correlation of pairs of variables with statistical adjustment to remove the statistical influence of the phylogenetic pattern. Pearson's correlations, unadjusted for phylogeny, were also calculated for comparison with phylogeny‐adjusted results. Cohen's (1988) criteria for effect sizes of associations were applied: small *r* = 0.1, medium *r* = 0.3, and large *r* ≥ 0.5.

## 3 RESULTS

The regression of maximum lifespan on adult female body mass was highly significant (*R* ^2^ = 0.59, *F* = 40.4, *df* = 1.28, *p* < .0001). The residuals of this analysis were fairly close to a Gaussian distribution (Shapiro–Wilk statistic = 0.98, *p* = .84) and were used as an estimate of mass‐independent lifespan. The positive association of maximum lifespan and body mass was strong and significant (*r* = 0.768, *t* = 6.4, *df* = 28, *p* < .0001).

Maximum lifespan had a strong associations with the phylogenetic pattern (ρ = 0.805, [credible intervals] CI_0.95_ = 0.284–0.907, *N* = 30). Body mass (and thus mass‐predicted lifespan) also had a strong association with phylogeny (ρ = 0.907, CI_0.95_ = 0.414–0.994, *N* = 30). Mass‐independent lifespan had at best a small association with phylogeny (ρ = 0.073, CI_0.95_ = 0.011–0.495, *N* = 30). Adult telomere length and TROC had trivial to small associations with the phylogeny (respectively; ρ = 0.073 and 0.062, CI_0.95_ = 0.017–0.497 and 0.018–0.568, *N* = 30).

**FIGURE 2:**
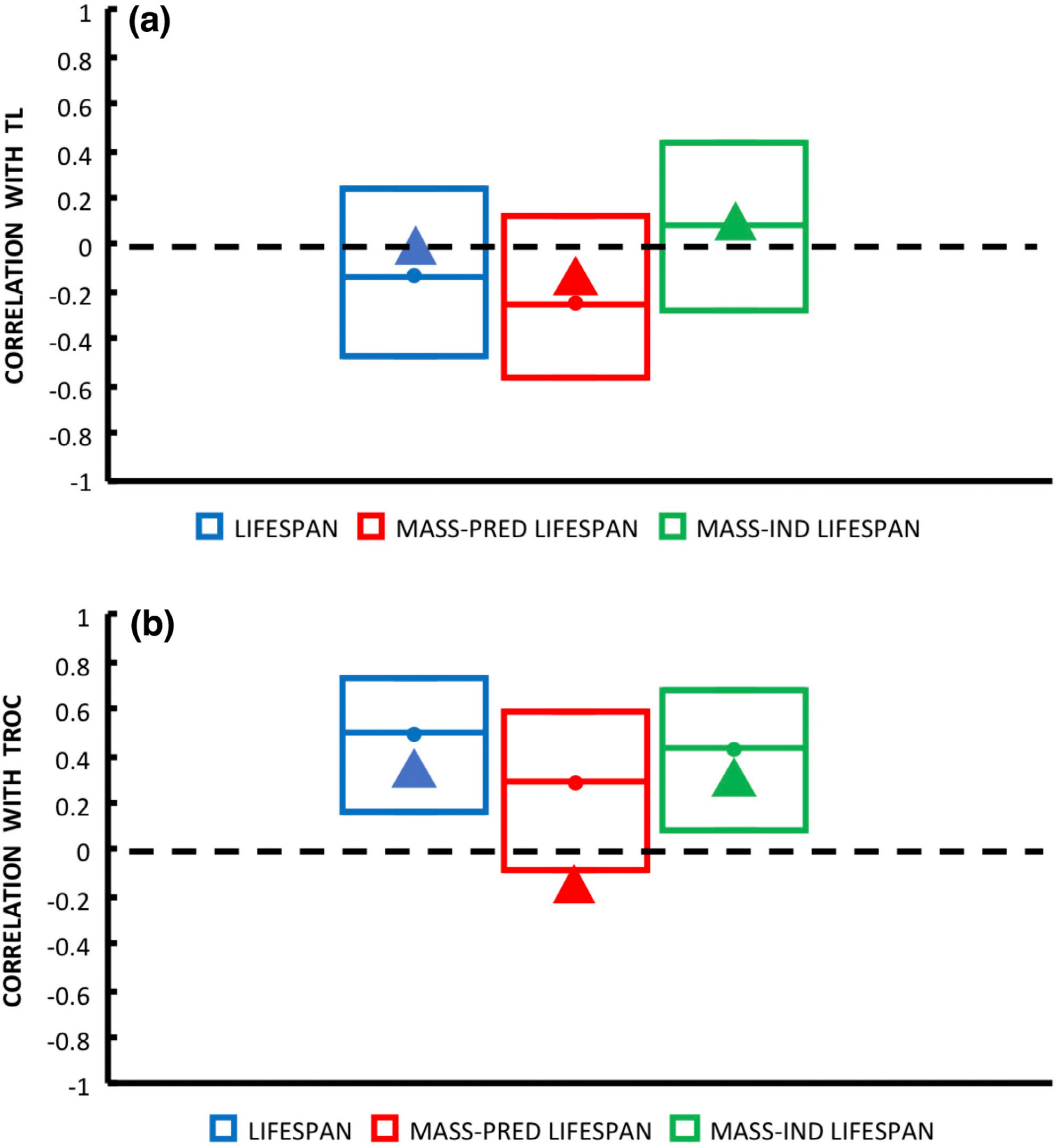
(a) Correlations of adult telomere length and three life‐history traits of 30 bird species: lifespan, mass‐predicted lifespan, and mass‐independent lifespan. (b) Correlations of telomere length rate of change (TROC) and the three aspects of longevity. “Lifespan” shows unadjusted correlations, “Mass‐Pred Lifespan” shows lifespan values predicted from the regression of lifespan on body size, and “Mass‐Ind Lifespan” shows the residuals of the regression of lifespan on body size. Pearson correlations are shown with horizontal center bars and 95% confidence intervals with high and low horizontal bars. Triangles show the phylogeny‐adjusted correlation values (none are significantly different from zero at the *p* < .05 level, except for TROC and mass‐independent lifespan). The horizontal black dashed lines show zero correlation.

Adult telomere length was not significantly correlated with maximum lifespan, mass‐predicted lifespan, or mass‐independent lifespan (Figure 2a). Once the phylogenetic pattern was taken into account statistically, adult telomere length exhibited trivial to moderate correlations with different estimates of lifespan (adjusted maximum lifespan, *r* = 0.004, CI_0.95_ = −0.356 – 0.394; adjusted mass‐predicted lifespan, *r* = −0.132, CI_0.95_ = −0.499 – 0.271; adjusted mass‐independent lifespan, *r* = 0.108, CI_0.95_ = −0.232 – 0.768; all *df* = 27, *p* > .20). It is noteworthy that the adults of large species tended to have shorter telomeres, but at a moderate correlation at best (unadjusted for phylogeny, *r* = −0.249, *t* = 1.4, *N* = 30, *p* = .18).

TROC was significantly positively correlated with lifespan (*r* = 0.497, *t* = 3.0, *df* = 28, *p* < .01), and this pattern was similar but not significant when the phylogenetic pattern was taken into account (adjusted *r* = 0.366, CI_0.95_ = −0.057 – 0.637, *df* = 27, *p* = .12) (Figure 2b). TROC exhibited a small to medium positive association with mass‐predicted lifespan that was not significant (*r* = 0.288, *t* = 1.6, *df* = 28, *p* = .12), and with phylogenetic “adjustment” this association turned slightly negative (adjusted *r* = −0.145, CI_0.95_ = −0.522 – 0.225, *df* = 27, *p* > .20). TROC and mass‐independent lifespan exhibited a moderate but significant positive correlation (*r* = 0.432, *t* = 2.5, *df* = 28, *p* = .02), and the effect size of this association was slightly lower but still significant when the phylogenetic pattern was taken into account statistically (adjusted *r* = 0.327, CI_0.95_ = 0.037–0.661, *df* = 27, *p* = .04).

## 4 DISCUSSION

Our first question concerned the association of life history and telomere variables with the phylogenetic pattern across 30 species of birds. Lifespan and body mass are well known to covary among bird species and exhibit strong phylogenetic constraint (e.g., Bennett & Owens, 2002; Criscuolo et al., 2021; Dantzer & Fletcher, 2015). Less is known of phylogenetic influences on telomere dynamics, but Tricola et al. (2018) suggested little phylogenetic influence on telomere length and a significant influence on TROC. We found the expected fairly strong association of phylogenetic pattern and lifespan and mass‐predicted lifespan, but a very weak association of the phylogeny with mass‐independent lifespan (long or short life at a given body size). Telomere length and the rate of decline in telomere length over time exhibited weak associations with the phylogeny, contrary to the suggestion of Tricola et al. (2018). Given the fairly strong association of phylogeny with lifespan and body mass, however, it seemed reasonable to account for the phylogenetic pattern statistically when evaluating associations of lifespan, body mass, and telomere dynamics.

Our second question was whether there was a strong association of lifespan and TROC, as suggested by Dantzer and Fletcher (2015) and Tricola et al. (2018). For this, we considered two aspects of longevity. Large animals live longer, as shown by a large number of studies on life‐history traits that scale with body size (e.g., Bennett & Owens, 2002; Dobson & Jouventin, 2007; Gaillard et al., 1989; Read & Harvey, 1989; Roff, 2002; Stearns, 1992). Larger animals take longer to grow to adult size and must allocate considerable resources and effort to maintaining their large number of cells. As such, the first question about longevity is whether it is associated with the overall size of an organism (Dobson, 2007). The second aspect of longevity is associated with the pace of life, along the so‐called “slow‐fast continuum” (Dobson & Oli, 2007; Gaillard et al., 1989). At a given body size, some species have greater maximum lifespan than others, and this may be associated with lower reproductive effort, and vice versa for short‐lived species. Thus, alternative life‐history tactics may be produced among species, at a given body size.

For the first aspect of lifespan that was associated with the size of the species, mass‐predicted lifespan had a small association with TROC that was not statistically significant, with or without statistical adjustment for the phylogenetic pattern (note that with adjustment for phylogeny, the sign of this correlation turned negative, Figure 2b). However, our lifespan variable that was independent of body size (viz., mass‐independent lifespan) had a moderate positive association with TROC as judged by its effect size, significant with or without statistical adjustment for phylogeny. These results suggest that TROC does not vary strongly with body size per se, but rather has at best a poor association with body size, such that longer‐lived species that are somewhat larger exhibited only slightly less telomere loss than somewhat smaller species. However, at a given body size, birds exhibited a stronger pattern of association of relative longevity (i.e., a slow pace‐of‐life) and TROC. Species with the longest lives for their body mass exhibited the slowest rate of loss of telomeres during life. Thus, the division of lifespan into two parts associated with different aspects of life histories revealed biologically meaningful patterns of varying strengths.

The analyses of Dantzer and Fletcher (2015), Tricola et al. (2018), Udroiu (2020), and Le Pepke and Eisenberg (2020) revealed a general pattern of positive association of longevity and TROC, but without testing for different underlying aspects of longevity. Our results revealed nuances to their conclusions: longevity and TROC showed no consistent pattern of change as body size increased, but rather there was a stronge pattern of longevity and TROC increasing together at a given body size. This pattern likely underlies the positive associations of longevity and TROC found by previous studies. Overall, the general agreement of unadjusted and phylogeny‐adjusted associations (Figure 2) may suggest that correlations in our results were little influenced by the historical evolutionary pattern reflected by the consensus phylogenetic tree.

Our final question was whether adult telomeres were shorter in the larger and longest‐lived species, as suggested by Gomes et al. (2011) and Le Pepke and Eisenberg (2021) for mammals. This latter study suggested that telomere length coevolved with body size, such that large species have short telomeres, and thus facilitated the evolution of long lifespans, notably via the use of cell replication senescence and the reduction of risks of cell immortalization (Risques & Promislow, 2018; Seluanov et al., 2018). On the other hand, Tricola et al. (2018) found a slight but non‐significant positive association of telomere length and maximum lifespan among 19 species of birds. While we found that both longevity and body mass followed the phylogenetic pattern fairly closely, telomere dynamics did not. Nonetheless, we found little evidence of larger species having shorter telomeres, with or without statistical adjustment for the influence of the phylogenetic pattern (Figure 2a). In the light of our results, postulating that large birds use replication senescence, as larger mammals do, as a mechanism favoring long lifespan is still an unanswered question. This begs the question of whether at least some bird species have evolved specific anti‐aging or anti‐cancer mechanisms that are similar to the telomere‐related control suggested for long‐lived mammalian species that weigh less than a kilogram (Gomes et al., 2011; Tian et al., 2018; but see Seluanov et al., 2018).

Comparative studies like the present one help to point out how aging mechanisms at the cell level may have coevolved with life histories among animal species. So far, as we have seen above, comparative studies have concluded that large body size and long lifespan have evolved with short telomeres and reduced loss of telomeres in mammals, or that longevity and reduced loss of telomeres (but not short telomeres) are matched in birds. This discrepancy might be attributed to the smaller range of sizes in birds, suggesting that if body size and the number of cells are the main constraint to the evolution of long telomeres, this may explain why birds show higher levels of telomere maintenance (e.g., via an enhanced telomerase expression) than mammals and long up‐to Mb telomeres (Delany et al., 2000; Monaghan, 2010). Our analysis that controlled for the effects of body size suggested that enhanced telomere maintenance has coevolved with longevity in birds independently of body size, and this differently, even in closely related species. This is, in addition to that of high glycemia and aerobic metabolism, a paradoxical association with avian longevity (Holmes & Harper, 2018), a new aging enigma that requires continued exploration in relation to species’ evolutionary histories.

## AUTHOR CONTRIBUTIONS

**F. Stephen Dobson:** Conceptualization (lead); formal analysis (lead); investigation (lead); writing – original draft (lead); writing – review and editing (equal). **Quentin Schull:** Formal analysis (supporting); writing – review and editing (equal). **François Criscuolo:** Data curation (lead); formal analysis (supporting); writing – review and editing (equal).

## FUNDING INFORMATION

The present study was supported by a USIAS fellowship and by a Gutenberg Chair awarded to F.S. Dobson. The study was also supported by the Agence Nationale Pour la Recherche, program AGEs, grant number ANR234061 to F. Criscuolo.

## Supporting information


Appendix S1

## Data Availability

Data file is provided in Appendix S1 and at Dryad (https://doi.org/10.5061/dryad.pnvx0k6rc).
